# Dietary supplementation with essential amino acids boosts the beneficial effects of rosuvastatin on mouse kidney

**DOI:** 10.1007/s00726-014-1772-5

**Published:** 2014-06-13

**Authors:** Giovanni Corsetti, Giuseppe D’Antona, Chiara Ruocco, Alessandra Stacchiotti, Claudia Romano, Laura Tedesco, Francesco Dioguardi, Rita Rezzani, Enzo Nisoli

**Affiliations:** 1 Division of Human Anatomy, Department of Clinical and Experimental Sciences, University of Brescia, 25123 Brescia, Italy; 2 Department of Medical Biotechnology and Translational Medicine, University of Milan, via Vanvitelli 32, 20129 Milan, Italy; 3 Department of Clinical Sciences and Community, University of Milan, 20122 Milan, Italy; 4 Department of Molecular Medicine, University of Pavia, 27100 Pavia, Italy

**Keywords:** Rosuvastatin, Essential amino acids, Kidney, Mitochondria, Mice

## Abstract

The effects of high-potency statins on renal function are controversial. To address the impact of statins on renal morpho-functional aspects, normotensive young mice were treated with rosuvastatin (Rvs). Moreover, because statins may impair mitochondrial function, mice received either dietary supplementation with an amino acid mixture enriched in essential amino acids (EAAm), which we previously demonstrated to increase mitochondrial biogenesis in muscle or an unsupplemented control diet for 1 month. Mitochondrial biogenesis and function, apoptosis, and insulin signaling pathway events were studied, primarily in cortical proximal tubules. By electron microscopy analysis, mitochondria were more abundant and more heterogeneous in size, with dense granules in the inner matrix, in Rvs- and Rvs plus EAAm-treated animals. Rvs administration increased protein kinase B and endothelial nitric oxide synthase phosphorylation, but the mammalian target of rapamycin signaling pathway was not affected. Rvs increased the expression of sirtuin 1, peroxisome proliferator-activated receptor γ coactivator-1α, cytochrome oxidase type IV, cytochrome c, and mitochondrial biogenesis markers. Levels of glucose-regulated protein 75 (Grp75), B-cell lymphoma 2, and cyclin-dependent kinase inhibitor 1 were increased in cortical proximal tubules, and expression of the endoplasmic reticulum–mitochondrial chaperone Grp78 was decreased. EAAm supplementation maintained or enhanced these changes. Rvs promotes mitochondrial biogenesis, with a probable anti-apoptotic effect. EAAm boosts these processes and may contribute to the efficient control of cellular energetics and survival in the mouse kidney. This suggests that appropriate nutritional interventions may enhance the beneficial actions of Rvs, and could potentially prevent chronic renal side effects.

## Introduction

According to the 2013 American College of Cardiology/American Heart Association Guideline on the Treatment of Blood Cholesterol, statins are the only class of drugs indicated for the prevention and treatment of atherosclerosis and cardiovascular disease (CVD) in subjects with increased risk (Stone et al. 2013). Statins are also known as 3-hydroxy-3-methylglutaryl-CoA (HMG-CoA) reductase inhibitors. HMG-CoA reductase is the rate-limiting enzyme in cholesterol synthesis, and its inhibition reduces pleiotropically the production of a number of molecules with inflammatory, endothelial, oxidant, and blood vessel tone properties (Susic et al. 2003; Bae et al. 2010; Fiore et al. 2011). Adverse effects and intolerance of statins depend on the specific prescribed molecule and on patient characteristics (Mancini et al. 2013). Adverse muscle, hepatic, and gastrointestinal effects have been extensively reported in the literature (Mansi et al. 2013), but controversy exists regarding the effects of high-potency statins on renal function. Statin-induced hematuria and proteinuria have been well established, and microalbuminuria is thought to be secondary to statin interference with the tubular reabsorption of albumin (van der Tol et al. 2012; Robles et al. 2013). In 36 studies of rosuvastatin (Rvs) involving more than 40,000 patients, renal impairment or renal failure was reported in only 536 participants (Stein et al. 2012). In addition, two meta-analyses that assessed the benefits and harms of statin use in patients with renal disease showed no deterioration, and rather a trend toward improvement or maintenance of renal function, with decreased CVD and mortality, in patients with chronic kidney disease (Upadhyay et al. 2012; Palmer et al. 2012).

Different potential mechanisms have been suggested as causing or contributing to statin side effects (Knauer et al. 2010). Mitochondrial mechanisms have been repeatedly implicated, particularly in muscle damage (Golomb and Evans 2008). Statins lead to dose-dependent reductions in coenzyme Q_10_ (De Pinieux et al. 1996), a key mitochondrial antioxidant and electron transport carrier that can bypass existing mitochondrial respiratory chain defects (Rosenfeldt et al. 2002). Several studies demonstrate that statins predispose to mitochondrial defects (Gambelli et al. 2004; Schick et al. 2007) in all users and, to a greater degree, in vulnerable individuals. A decreased Q_10_ content was accompanied by a decreased maximal oxidative phosphorylation (OXPHOS) capacity in simvastatin-treated patients (Larsen et al. 2013). Moreover, dose-dependent reductions in coenzyme Q_10_ can reduce cell energy, promote oxidation or apoptosis, and unmask silent mitochondrial defects (Lenaz et al. 2002). It is plausible that these findings partly explain the muscle pain and exercise intolerance that many patients experience with their statin treatment.

Extensive evidence supports the efficacy of dietary supplementation with a balanced amino acid mixture enriched in essential amino acids (EAAm) in clinical disorders characterized by deficits of energy production, including ageing, type 2 diabetes, heart failure, alcohol consumption, and renal failure (Cano et al. 2006; Valerio et al. 2011; Corsetti et al. 2012). It is widely accepted that the kidneys play a major role in amino acid metabolism and, partially, in the regulation of amino acid plasma levels. About 97 % of the filtered amino acids are actively reabsorbed by renal tubules, and amino acids affect several kidney physiological processes depending on their quality and quantity (Silbernagl 1988). Among others, arginine, the substrate of nitric oxide synthase (NOS) and thus the precursor of NO synthesis (Andrew and Myer 1999), plays key roles in renal vessel and tubular functions (Rajapaskse and Mattson 2013). Arginine ingestion or systemic administration increases renal blood flow and glomerular filtration rate in humans (Kano et al. 1992), possibly by displacement of asymmetric-di-methyl-arginine (ADMA), the endogenous inhibitor of endothelial nitric oxide synthase (eNOS) (MacAllister and Vallance 1994). Furthermore, renal eNOS is present not only in endothelial cells but also in the cells of the collecting ducts and macula densa, where NO regulates tubulo-glomerular feedback and renin secretion (MacAllister and Vallance 1994). The mTOR-AKT-eNOS pathway is known to be the cellular sensor of amino acid availability (Zoncu et al. 2011). Signaling via mTOR has been implicated in proliferative kidney diseases with an increased phosphorylation of downstream targets, including ribosomal protein S6 (Shillingford et al. 2006).

Notably, the EAAs leucine, lysine and phenylalanine act similarly to insulin and suppress proteolysis of circulating and renal peptides. Supplementation with this EAAm was found to slow renal senescence in rodents (Corsetti, et al. 2010) and in hemodialysis patients (Bolasco et al. 2011). We have recently demonstrated that this specific amino acid formula restores mitochondrial function and antioxidant responses through eNOS expression, with reduced inflammatory processes, in the organs and tissues of aged rodents (D’Antona et al. 2010; Corsetti et al. 2011). Although lacking arginine, the EAAm promotes arginine synthesis (Rondanelli et al. 2011).

The present work aimed to study firstly the effects of long-term treatment with Rvs on the morphological and immunohistochemical aspects of the kidney in young mice, and secondly the impact of EAAm supplementation on the renal effects of Rvs, mainly focusing on mitochondrial adaptive processes.

## Materials and methods

### Animals and treatments

C57BL/6 male mice (2 months old, *n* = 36, Charles River, Calco, Italy) were housed in a quiet room with controlled temperature and humidity. A 12 h/12 h light/dark cycle was maintained (lights on from 7 a.m. to 7 p.m.). The experimental protocol was conducted in accordance with European Community guidelines and the Italian Ministry of Health, complied with The National Animal Protection Guidelines, and was approved by the Institutional Animal Ethical Committee.

Animals were given unrestricted access to a standard diet (4.3 % fat, 18.8 % protein, 76.9 % carbohydrate, Laboratorio Dottori Piccioni; for information about dietary ingredients and actual composition of the diet see Table 1) and tap water, and were treated for up to 1 month with Rvs (20 mg/kg/day, *n* = 10 animals), Rvs plus EAAm (1.5 mg/g body weight, *n* = 10 animals), or EAAm alone (1.5 mg/g body weight, *n* = 8 animals) via drinking water. A control group received drinking water without any drugs or supplement (CTRL; *n* = 8 animals). EAAm (composition, relative percentage, and dietary intake of each amino acid are reported in Table 2) was dissolved in tap water in quantities determined by calculating average daily drinking for 2 weeks before starting treatment (approximately 6 ml as in Bachmanov et al. 2002) and stored at 4 °C before daily administration. EAAm concentrations were previously found to be active in rodents and mimic the recommended daily dose for humans (see Pellegrino et al. 2005; D’Antona et al. 2010). The Rvs dosage selected in this study is comparable to those adopted in other studies conducted to prove its ability to lower blood cholesterol (Enomoto et al. 2009).

**Table 1:** Dietary ingredients, amino acid content and actual composition of the diet

Macronutrient composition			
Nutrient	%	Amino acid	g/100 g
Raw proteins	18.8	Arginine	1.15
		Histidine	0.48
		Isoleucine	0.80
		Leucine	1.50
		Lysine	1.05
		Methionine + cysteine	0.75
		Phenylalanine	0.90
		Threonine	0.75
		Tryptophan	0.23
		Valine	1.00
Crude fat	4.3		
Crude cellulose	3.8		
Crude ash	7.7		
Humidity	12		
**Micronutrient composition** (mg/kg)			
Vitamin A	12,000 UI	Folic acid	2
Vitamin D	1,000 UI	Choline cloride	1,000
Vitamin E	40	Biotin	0.1
Vitamin B1	8	Iron	100
Vitamin B2	10	Cobalt	0.25
Vitamin B6	10	Copper	3
Pantothenic acid	15	Manganese	55
Vitamin K	1	Iodine	0.8
Vitamin PP	40	Zinc	50
Vitamin B12	0.02	DL-methionine	500

Amino acid contents are reported as g/100 g mice food. Methionine was supplemented to the basic mixture

**Table 2:** Composition, relative percentage and dietary intake of EAAm

Amino acid	Molecular mass (g/mol)	Percentage (%)	Dietary intake (g/kg/day)
Leucine	131.2	30.5	0.457
Lysine	142.2	13.2	0.198
Isoleucine	131.2	15.6	0.234
Valine	117.1	19.6	0.294
Threonine	119.1	10.8	0.162
Cysteine	121.2	4.4	0.066
Histidine	155.2	2.7	0.040
Phenylalanine	165.2	1.6	0.024
Methionine	149.2	1	0.015
Tyrosine	181.2	0.4	0.006
Tryptophan	204.2	0.2	0.003

Body weight, kidney weight, and average daily food and water consumption are reported in Table 3. Interestingly, the dietary EAAm supplementation induced a non-statistically significant reduction of food consumption in Rvs-treated and Rvs-untreated mice in comparison to the respective controls. This reduction led to slight changes in daily protein intake (CTRL: 0.76 ± 0.07 g/day; EAAm: 0.65 ± 0.24 g/day; Rvs: 0.76 ± 0.11 g/day; Rvs plus EAAm: 0.75 ± 0.12 g/day).

**Table 3:** Body weight, kidney weight, feeding, and water consumption

	Control	EAAm	Rvs	Rvs + EAAm
Body weight (g)	28.25 ± 1.1	27.95 ± 0.9	28.12 ± 1.4	27.43 ± 1.2
Kidney weight (g)	1.02 ± 0.14	1.11 ± 0.17	1.09 ± 0.1	1.05 ± 0.09
Food intake (g)	4.06 ± 0.4	3.92 ± 0.12	4.1 ± 0.5	3.96 ± 0.7
Water intake (g)	6.1 ± 1.3	5.8 ± 1.4	6.2 ± 0.8	5.9 ± 1.1

Measurements were done in 8–10 animals per group. Values represent mean ± SEM. Statistical analysis did not show any differences between groups

At the end of the study treatment, mice were killed under deep ether anesthesia at 09.00–10.00 a.m., blood samples were collected, and the kidneys were quickly removed and placed in an ice-cold saline solution. Samples used for histochemical analysis were mounted in Tissue-Tek^®^ OCT (Sakura Finetek Europe, Alphen aan den Rijn, The Netherlands) embedding medium, before being frozen in liquid nitrogen and stored at –80 °C. Samples used for immunohistochemical analysis and morphometry were fixed in buffered 4% formaldehyde and stored at 4 °C for 24 h, and then paraffin was added. Samples used for mRNA, mtDNA and protein analysis were quickly frozen in liquid nitrogen and stored at –80 °C.

### Plasma amino acid levels

Amino acid analysis was performed with the method previously described slightly modified (Aquilani et al. 2000). Briefly the concentration of free amino acids in plasma was determined by means of the AminoQuant II amino acid analyser based on the HP Amino Quant 1090L HPLC system with fully automated precolumn derivatization using both ortho-phtalaldehyde (OPA, for amino acids containing primary amine groups) and 9-fluorenylmethylchloroformate (FMOC, for amino acids containing secondary amine groups, i.e., proline and hydroxyproline) reaction chemistries according to the manufacturer’s protocol. Derivatized amino acids were separated by reverse-phase HPLC on hypersil ODS 250 × 2.1 mm, 5 μm column thermostatted at 40 °C and absorbance was recorded at 338 excitation and 390 nm emission for OPA and 262 nm excitation and 324 nm emission for FMOC, using a diode array detector. The procedure used was as follows: 500 μL samples of plasma were deproteinized by adding 250 μL of 0.5 N HCI and, after centrifugation at 5,000*g* for 10 min at 5 °C, the supernatant was concentrated up to 200 μL under a nitrogen stream and further filtered on a 0.22 μm Spin-X filter. Aliquots (1 μL each) were automatically transferred to the reaction coil and derivatized. The remaining deproteinized serum was stored at 20 °C. Analyses were performed in duplicate, and the value reported for each amino acid was the mean of two independent determinations. The amino acid plasma concentrations were expressed as pmol/μl.

### Glomeruli morphometry

All measurements were obtained by a blind observer using standard morphometric techniques on periodic acid–Schiff (PAS) stained sections (Corsetti et al. 1998). The number of glomeruli (N*glo*), the mean area of glomeruli (A*glo*), and the total area of the renal parenchyma (A*tot*) were measured from thick sections stained with epoxy tissue stain. The ratio between A*glo* and A*tot* (A*glo*/A*tot*), and the number of glomeruli per unit area, also called glomerular density (N*glo*/mm^2^), were calculated.

### Histochemistry and electron microscopy

Collagen deposition and fibrosis were evaluated by sirius red staining using a modified picro-sirius procedure, as previously described (Dayan et al. 1989). Briefly, frozen slices were serially sectioned at 5 μm and stained with sirius red, and collagen fibers were detected by polarized light microscopy (Olympus, Hamburg, Germany). Type I collagen (newly-formed) fibers appear yellow–red, and type III collagen (constitutive) fibers appear green. For transmission electron microscopy analysis, renal cortex pieces were immersed in 2.5 % glutaraldehyde for 3 h at 4 °C, post-fixed in 1 % osmium tetroxide, and embedded in Epon 812 epoxic mixture as previously reported (Stacchiotti et al. 2011).

### Immunohistochemistry

Sections were incubated overnight with primary antibodies. Anti-eNOS and anti-Bcl-2 polyclonal antibodies (both from Santa Cruz, CA, USA) were diluted 1:100 in PBS. Anti-Grp75 and anti-Grp78 (both from Stressgen, Vinci-Biochem, Vinci, Italy) polyclonal antibodies were diluted 1:300 in PBS. The sections were processed in accordance with the manufacturers’ protocols, visualized with a rabbit ABC-peroxidase staining system kit (Santa Cruz), and dehydrated and mounted with Distyrene Plasticizer Xylene (DPX). The reaction product was visualized using 0.3 % H_2_O_2_ and 3,3′-diaminobenzidine (DAB) at room temperature with positive staining appearing as a brownish color. Some sections were incubated with isotype-matched IgGs instead of primary antibodies as controls. Similar results were obtained when experiments were performed using the peroxidase–anti-peroxidase detection system, confirming that the presence of endogenous biotin did not lead to misinterpretation of the immunostaining data. Each set of experiments was performed in triplicate under the same experimental conditions. The intensity of histochemical and immunohistochemical staining was measured using an optical Olympus BX50 microscope equipped with an image analysis program (Image Pro Plus™ 4.5.1, Immagini & Computer, Milan, Italy) and was then analyzed quantitatively. The integrated optical density (IOD) was calculated for arbitrary areas by observing ten fields in each sample with a 40× objective. Data were pooled, mean values were calculated, and the results of each group were statistically compared.

### Gene expression and mtDNA quantification

RNA was isolated from kidneys using the RNA easy Mini Kit (Qiagen, Milan, Italy) and cDNA was synthesized using iScript cDNA Synthesis Kit (Bio-Rad Laboratories, Segrate, Italy), as described by D’Antona et al. (2010). For the evaluation of mtDNA, total DNA was extracted with QIAamp DNA extraction kit (Qiagen). The mRNA levels and mtDNA amount were measured by quantitative Real-Time PCR in triplicate, with iTaq Universal SYBR Green SuperMix (Bio-Rad Laboratories) on a CFX Connect Real-Time PCR System (Bio-Rad Laboratories). Primers were designed using Primer3 (version 0.4.0) software and are shown in Table 4. The cycle number at which the various transcripts were detectable (threshold cycle, CT) was compared with housekeeping CT, referred to as ΔCT. The gene relative level was expressed as 2^−(ΔΔCT)^, in which ΔΔCT corresponded to the difference between the ΔCT of either treatment group and the ΔCT of the control group.

**Table 4:** Primers used for quantitative PCR analysis

Gene	ID	Primer sequences		PCR product	*T*_a_ (°C)
PGC1-α	NM_008904.2	*Sense*	5′-ACTATGAATCAAGCCACTACAGAC-3′	148 bp	60
		*Antisense*	5′-TTCATCCCTCTTGAGCCTTTCG-3′		
Tfam	NM_009360.4	*Sense*	5′-AAGACCTCGTTCAGCATATAACATT-3′	104 bp	60
		*Antisense*	5′-TTTTCCAAGCCTCATTTACAAGC-3′		
NRF-1	NM_001164226.1	*Sense*	5′-ACAGATAGTCCTGTCTGGGGAAA-3′	99 bp	60
		*Antisense*	5′-TGGTACATGCTCACAGGGATCT-3′		
CDKN1A	NM_007669.4	*Sense*	5′-TTGCACTCTGGTGTCTGAGC-3′	127 bp	60
		*Antisense*	5′-GGGCACTTCAGGGTTTTCTC-3′		
mtDNA	NC_005089.1	*Sense*	5′-ACATGCAAACCTCCATAGACCGG-3′	131 bp	60
		*Antisense*	5′-TCACTGCTGAGTCCCGTGGG-3′		
GAPDH	NM_008084	*Sense*	5′-AACTTTGGCATTGTGGAAGG-3′	183 bp	60
		*Antisense*	5′-ACACATTGGGGGTAGGAACA-3′		

*T*_*a*_ annealing temperature

### Immunoblot analysis of mitochondrial markers

Protein extracts were obtained from kidneys using T-PER Mammalian Protein Extraction Reagent (Pierce, Thermo Scientific, Rockford, USA), as indicated by the manufacturer, in the presence of a cocktail of protease and phosphatase inhibitors (Sigma-Aldrich, Milan, Italy). Protein content was determined with bicinchoninic acid protein assays (BCA, Pierce, Euroclone, Milan, Italy). An appropriate amount of protein was run on SDS-PAGE under reducing conditions for immunoblotting. The separated proteins were then semi-dry transferred to a nitrocellulose membrane (Bio-Rad Laboratories) and proteins of interest were revealed with specific antibodies: anti-p-S6 (Ser235/236), anti-S6, anti-p-AKT (Ser473), anti-AKT, anti-p-eNOS (Ser1177), anti-eNOS, anti-COX IV, anti-Cyt c, and anti-Grp78 (all from Cell Signaling, Euroclone, Milan, Italy); anti-SIRT1, anti-Grp75, and anti-Bcl-2 (all from Santa Cruz); and anti-PGC-1α (Abcam, Cambridge, UK) each at 1:1,000 dilution. Anti-β-Actin (1:10,000; Cell Signaling) and anti-Vinculin (1:10,000; Sigma-Aldrich) were used as loading controls. Immunostaining was detected using horseradish peroxidase conjugated anti-rabbit or anti-mouse immunoglobulin for 1 h at room temperature (Tedesco et al. 2010). Amounts of each protein were measured using SuperSignal Substrate (Pierce) and densitometrically quantified with an IMAGEJ software image analyzer.

### Statistical analysis

Morphometric data were expressed as mean ± standard deviation (SD) or standard error of the mean (SEM) unless otherwise stated. Statistical analysis was performed using two-way ANOVA followed by Bonferroni post hoc test for multiple comparisons (GraphPad Prism, CA). Statistical significance was set at *p* < 0.05.

## Results

### Plasma amino acid levels

Table 5 shows the plasma amino acid levels in the different groups of mice. In particular, glycine, leucine, lysine, and threonine were higher, in plasma of EAAm-treated mice compared to untreated animals. Accordingly to the results obtained by Trupp et al. (2012) in humans treated with simvastatin, Rvs increased threonine, alanine and valine levels compared to untreated animals. Finally, changes were seen in Rvs plus EAAm group, with increased levels of alanine, glycine, threonine, and valine compared to untreated animals. Interestingly, the only significant interaction between Rvs and EAAm treatment was observed for alanine. The plasma levels of this amino acid were higher in comparison with EAAm and Rvs alone thus suggesting an additive effect of the treatments. Overall, the effects of EAAm and Rvs on plasma amino acids suggest that changes in certain circulating amino acids may contribute to the observed effects in the mouse kidney.

**Table 5:** Plasma amino acid profile after Rvs and/or amino acid treatment in mice

	Control	EAAm	Rvs	Rvs + EAAm
	Mean ± SEM	Mean ± SEM	Mean ± SEM	Mean ± SEM
Alanine	372 ± 16	351 ± 15^††, §^	453 ± 23*	451 ± 29*, ‡
Arginine	243 ± 27	220 ± 22	266 ± 46	196 ± 17
Asparagine	21 ± 1	19 ± 1	19 ± 1	21 ± 2
Aspartic acid	16 ± 2	19 ± 3	18 ± 3	23 ± 3
Citrulline	70 ± 6	65 ± 5	69 ± 7	58 ± 7
Cysteine	14 ± 1	16 ± 1	16 ± 1	16 ± 1
Glutamic acid	67 ± 4	78 ± 5	75 ± 4	73 ± 4
Glutamine	627 ± 35	546 ± 24	594 ± 19	600 ± 14
Glycine	203 ± 4	258 ± 13**	224 ± 15	270 ± 12***
Histidine	46 ± 1	47 ± 2	37 ± 5	39 ± 4
1Met-histidine	8 ± 1	8 ± 1	9 ± 1	9 ± 1
Isoleucine	66 ± 10	85 ± 6	72 ± 8	89 ± 5
Leucine	122 ± 9	167 ± 18*	153 ± 10	151 ± 10
Lysine	89 ± 22	162 ± 22*, †	93 ± 15	120 ± 14
Methionine	59 ± 4	55 ± 7	56 ± 9	42 ± 2
Ornitine	54 ± 14	58 ± 12	69 ± 11	80 ± 14
Phenylalanine	53 ± 5	69 ± 9	70 ± 11	73 ± 4
Proline	90 ± 5	100 ± 7	105 ± 9	107 ± 13
Hydroxyproline	102 ± 36	104 ± 41	100 ± 14	87 ± 10
Serine	123 ± 6	118 ± 8	120 ± 8	122 ± 8
Threonine	123 ± 10	159 ± 9*	164 ± 8*	160 ± 9*
Tryptophan	93 ± 7	93 ± 7	95 ± 7	95 ± 7
Tyrosine	86 ± 7	85 ± 8	92 ± 10	92 ± 7
Valine	142 ± 11	155^†^ ± 14	218 ± 17**	215 ± 18**
Sum AA	2,861 ± 82	3,025 ± 120	3,142 ± 63	3,203 ± 79

Plasma amino acid profile after Rvs and/or amino acid treatment in mice. Plasma concentrations of individual amino acids (pmol/μl) were measured in control mice fed *ad libitum* (CTRL; *n* = 8), dietary supplemented with amino acid mixture (EAAm *n* = 8), treated with rosuvastatin (Rvs *n* = 8) and rosuvastatin plus EAAm (Rvs + EAAm *n* = 8). Sum AA, total amino acids. Statistical significance was tested by two-way ANOVA followed by Bonferroni post hoc test for multiple comparison

* Indicates significantly different vs Control values (* = *p* < 0.05, ** = *p* < 0.01, *** = *p* < 0.001);^ †^, indicates significantly different vs Rsv treatment values (^†^ = *p* < 0.05, ^††^ = *p* < 0.01); §, indicates significantly different *vs* Rvs + EAAm treatment values, *p* < 0.05; ^‡^, indicates significant interaction *p* < 0.001

### Histology and ultrastructure analysis

**Fig. 1:**
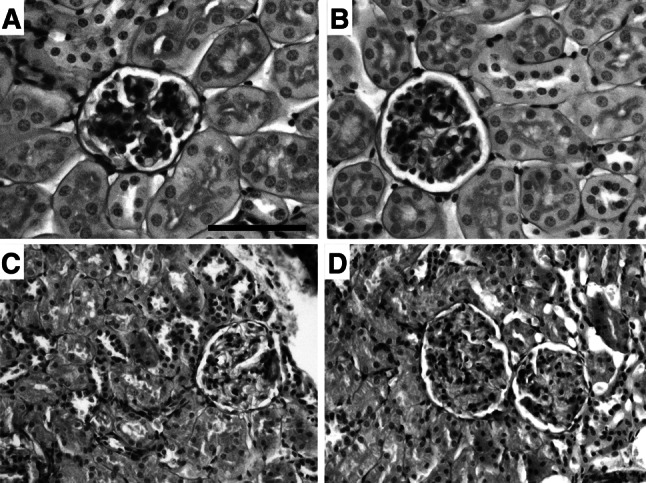
Renal histological analysis. PAS staining in untreated (CTRL) (**a**), EAAm (**b**), Rvs (**c**), and Rvs + EAAm (**d**) groups. Histological evaluation revealed no detectable renal damage, fibrosis or other alteration in any of the groups or renal compartments. Experiments were performed in 8–10 animals per group. *EAAm* amino acid mixture enriched in essential amino acids, *Rvs* rosuvastatin. *Bar* 50 μm

**Fig. 2:**
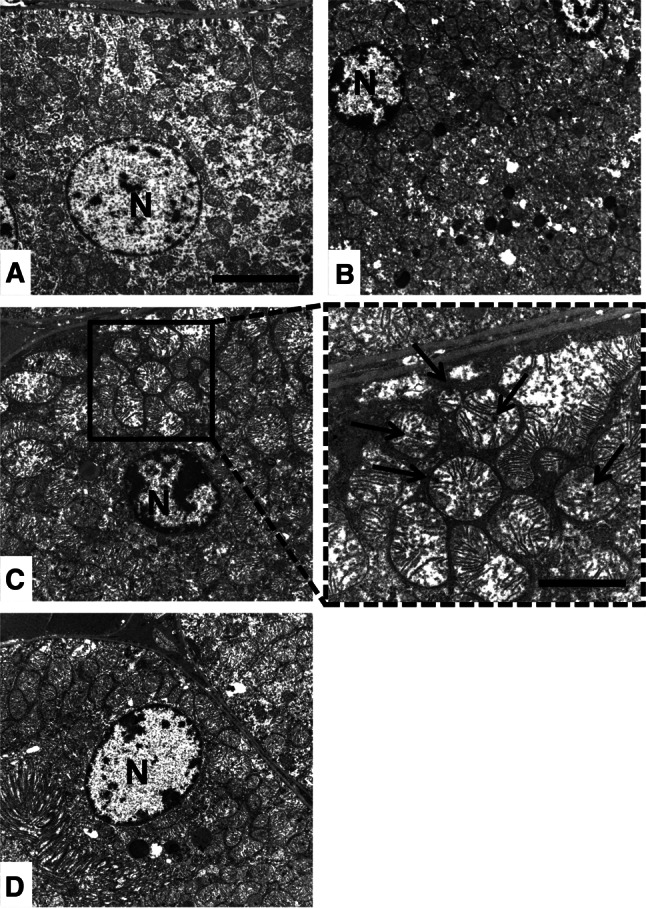
Electron microscopy analysis of mouse kidneys. **a** Untreated mice (CTRL). **b** EAAm-treated mice; the mitochondria were abundant, round and with dense matrix. *Bar* 5 μm. **c** Rvs-treated animals; mitochondria were more heterogeneous, elongated and scattered around the nucleus. *Bar* 5 μm. *Inset* higher magnification; irregular cristae and electron-dense granules in the mitochondrial matrix resembling calcium deposits (*black arrows*) were evident. *Bar* 2 μm. **d** Rvs + EAAm treatment. Similar features as in panel **c**. Experiments were performed in 8–10 animals per group. *EAAm* amino acid mixture enriched in essential amino acids, *Rvs* rosuvastatin, *N* nucleus. *Bar* 5 μm

Body weight, feeding, and water consumption in each group of mice are shown in Table 3. No differences were evident between Rvs- and Rvs plus EAAm-treated mice, or between these treated animals and the untreated controls. Kidney weight, a parameter related to renal function, did not differ between groups. Histological evaluation after staining with hematoxylin/eosin, sirius red (for collagen detection), or PAS (for detecting polysaccharides, including glycogen, and mucosubstances, including glycoproteins, glycolipids and mucins) did not reveal damage, fibrosis, or other evident changes in any renal compartment (Fig. 1 and data not shown). The number of renal glomeruli (N*glo*/mm^2^) and the ratios between cross-sectional glomerular areas and total area (A*glo*/A*tot)* were not statistically different between the experimental groups (Table 6). Ultrastructural analysis of the proximal tubular cells, which are characterized by a high OXPHOS mitochondrial activity (Szeto et al. 2011) and which are actively involved in Rvs excretion (Verhulst et al. 2008), showed abundant, round-shaped mitochondria, particularly in the EAAm-supplemented group (Fig. 2). Heterogeneous and elongated mitochondria, with multiform cristae and electron-dense granules in the matrix, were more evident around the nuclei of these cells both in Rvs- and Rvs plus EAAm-treated mice when compared to untreated ones (Fig. 2).

**Table 6:** Glomeruli morphometry

	Control	EAAm	Rvs	Rvs + EAAm
N*glo*/mm^2^	15.12 ± 1.51	16.4 ± 0.9	15.01 ± 1.77	15.98 ± 1.48
A*glo*/A*tot*	0.06 ± 0.02	0.06 ± 0.01	0.04 ± 0.02	0.05 ± 0.02

N*glo*/mm^2^, density of glomeruli; A*glo*/A*tot*, ratio between glomerular area and total parenchymal area. Values were expressed as mean ± SEM. Measurements were done in 8–10 animals per group

### Rosuvastatin alone or in combination with EAAm promotes mitochondrial biogenesis and function in mouse kidneys

**Fig. 3:**
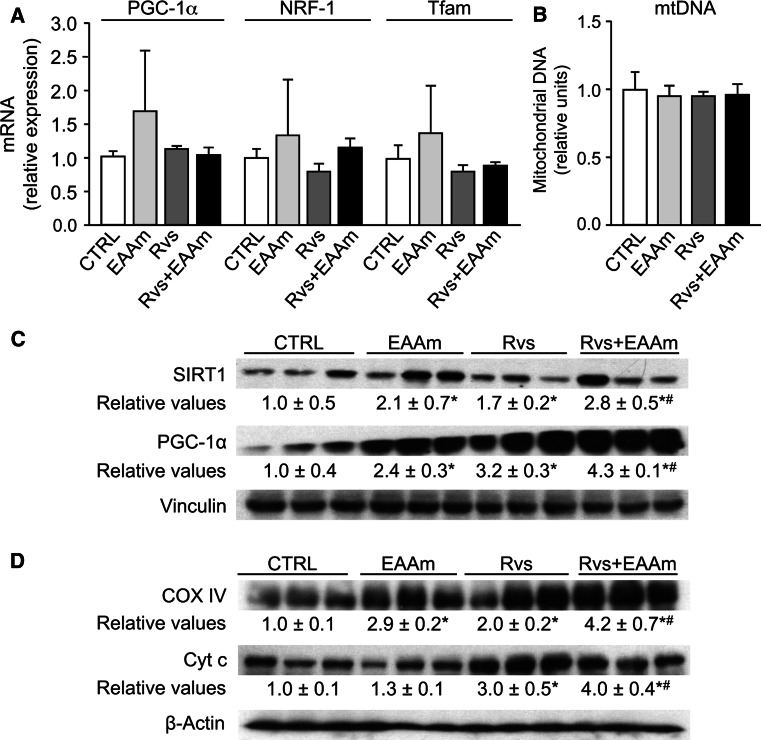
Molecular markers of mitochondrial biogenesis in mouse kidneys. **a** mRNA levels of mitochondrial biogenesis markers and **b** mtDNA amounts in the kidneys of untreated (CTRL) and treated groups analyzed by quantitative RT-PCR. The cycle number at which the transcripts were detectable was compared to that of GAPDH and expressed as relative expression versus controls, taken as 1.0. The experiments were performed in 8–10 animals per group and data are expressed as mean ± SEM. **c**, **d** Effects of treatments with Rvs and EAAm, either alone or in combination, on SIRT1, PGC-1α, COX IV, and Cyt c protein levels measured by immunoblot analysis. The Western blot images are representative of separate experiments done in 8–10 animals per group. The relative values were detected by densitometric analysis, relative to either vinculin or β-actin levels, with untreated (CTRL) mice given a value of 1.0. Data are expressed as mean ± SEM. *p < 0.05 vs untreated (CTRL) mice; ^#^ *p* < 0.05 vs Rvs-treated mice

The effects of Rvs alone or in combination with EAAm on mitochondrial biogenesis were investigated in kidney homogenates from treated and untreated animals. Figure 3a, b shows that neither treatment changed the mRNA levels of peroxisome proliferator-activated receptor γ coactivator 1α (PGC-1α), nuclear respiratory factor 1 (NRF-1), mitochondrial transcription factor A (Tfam), nor the amount of mtDNA (an index of mitochondrial mass) in the kidneys. Both Rvs and EAAm increased the protein levels of sirtuin 1 (SIRT1), a highly conserved class III histone deacetylase sensitive to nutritional status that is able to activate PGC-1α (Fig. 3c). Protein levels of PGC-1α, which acts as a regulator of mitochondrial biogenesis, were also increased (Fig. 3c). Notably, Rvs plus EAAm showed an additive effect on SIRT1 and PGC-1α protein levels (Fig. 3c). Similar results were obtained with COX IV and Cyt c, respiratory proteins that are an indirect index of mitochondrial function. EAAm alone had a small effect on Cyt c levels (Fig. 3d). These results suggest that Rvs promotes the expression of some markers of mitochondrial biogenesis and that EAAm supplementation reinforces this effect.

### Rosuvastatin stimulates AKT and eNOS phosphorylation in mouse kidneys

**Fig. 4:**
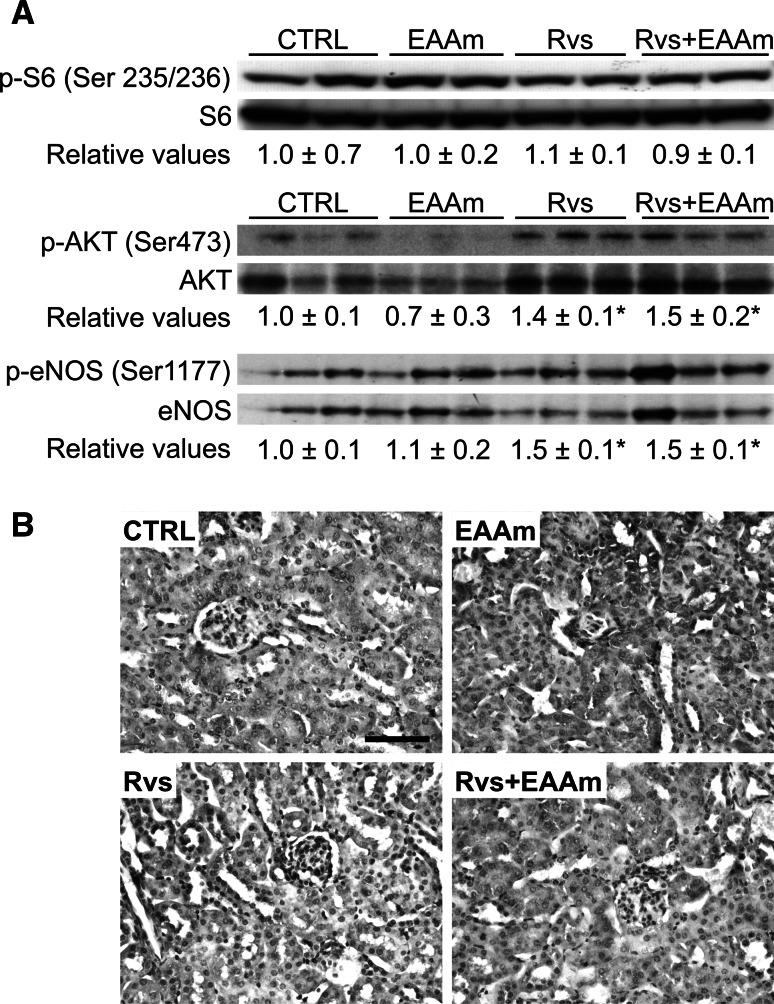
Rosuvastatin and amino acids affect the AKT-eNOS pathway in mouse kidneys. **a** Rvs alone or in combination with EAAm activates AKT and eNOS, but not mTOR, in kidneys. The phosphorylated S6, AKT and eNOS protein levels were analyzed by immunoblot, and the relative values were detected by densitometric analysis, relative to S6, AKT, or eNOS total protein levels, when control measurement was given a value of 1.0. The Western blot images are representative of separate experiments performed in 8–10 animals per group. Data are expressed as mean ± SEM. *p < 0.05 vs untreated mice (CTRL). **b** eNOS immunohistochemical analysis. *EAAm* amino acid mixture enriched in essential amino acids, *Rvs* rosuvastatin. *Bar* 50 μm

To investigate the signaling mechanism(s) involved in the effects of Rvs and EAAm on renal mitochondria, we analyzed the mTOR-AKT-eNOS pathway. To determine whether Rvs and EAAm affected mTOR signaling in mice, kidney lysates were prepared and the amounts of phosphorylated S6 (p-S6) were determined by immunoblot analysis (Fig. 4a). The level of p-S6, normalized to total S6 (pS6/S6), was unchanged by either Rvs or EAAm, alone or in combination. In accordance with previous results (Bussolati et al. 2005; Ito et al. 2010), Rvs activated protein kinase AKT through increased Ser473-AKT phosphorylation (Fig. 4a). This effect was not strengthened by EAAm, which alone was unable to modify AKT phosphorylation (Fig. 4a). Neither Rvs nor EAAm, or their combination, promoted phosphorylation of AKT in Thr308 (data not shown). Activated AKT phosphorylates multiple targets, including eNOS. Ser117 phosphorylation of eNOS increases nitric oxide production (Kureishi et al. 2000). Accordingly, Rvs increased Ser1177 p-eNOS levels in the kidney, while EAAm did not change either basal or Rvs-induced eNOS phosphorylation (Fig. 4a). Specific immunohistochemical analysis showed a moderate eNOS immunostaining uniformly distributed in the cortical tubular cells of untreated and EAAm-supplemented mice (Fig. 4b; Table 7). The staining intensity was weakly decreased in the kidney of Rvs-treated animals, but eNOS was markedly increased when Rvs was used in combination with EAAm, particularly in the proximal tubular cells (Fig. 4b; Table 7). Faint and sparse staining for inducible and neuronal NOS (iNOS and nNOS) was observed in the kidneys of each group (data not shown).

**Table 7:** Immunohistochemical measurements

	Control	EAAm	Rvs	Rvs + EAAm
eNOS	20.5 ± 2.1	30.1 ± 2.3^†^	18.3 ± 1.2*	25.9 ± 1.7^#^
Grp75	32.3 ± 3.1	30.5 ± 2.8	18.2 ± 3.7^†, ^*	21.1 ± 3.2
Grp78	18.6 ± 2.6	20.4 ± 3.1	37 ± 4.4^†,^ *	30.2 ± 4.6
Bcl-2	9.1 ± 0.9	19.4 ± 1.2^†^	18.3 ± 2.7^†^	25.2 ± 1.8^†, #, ^*

Integrated optical density (IOD) values (±SEM) of immunohistochemical staining in each group. Measurements were done in 8–10 animals per group

† * p* < 0.05 vs Control group * p < 0.01 vs EAAm group; ^#^ *p* < 0.05 vs rosuvastatin group, two-way ANOVA–Bonferroni post hoc test

### Rosuvastatin regulates the physiology of mitochondria and endoplasmic reticulum in mouse kidneys

**Fig. 5:**
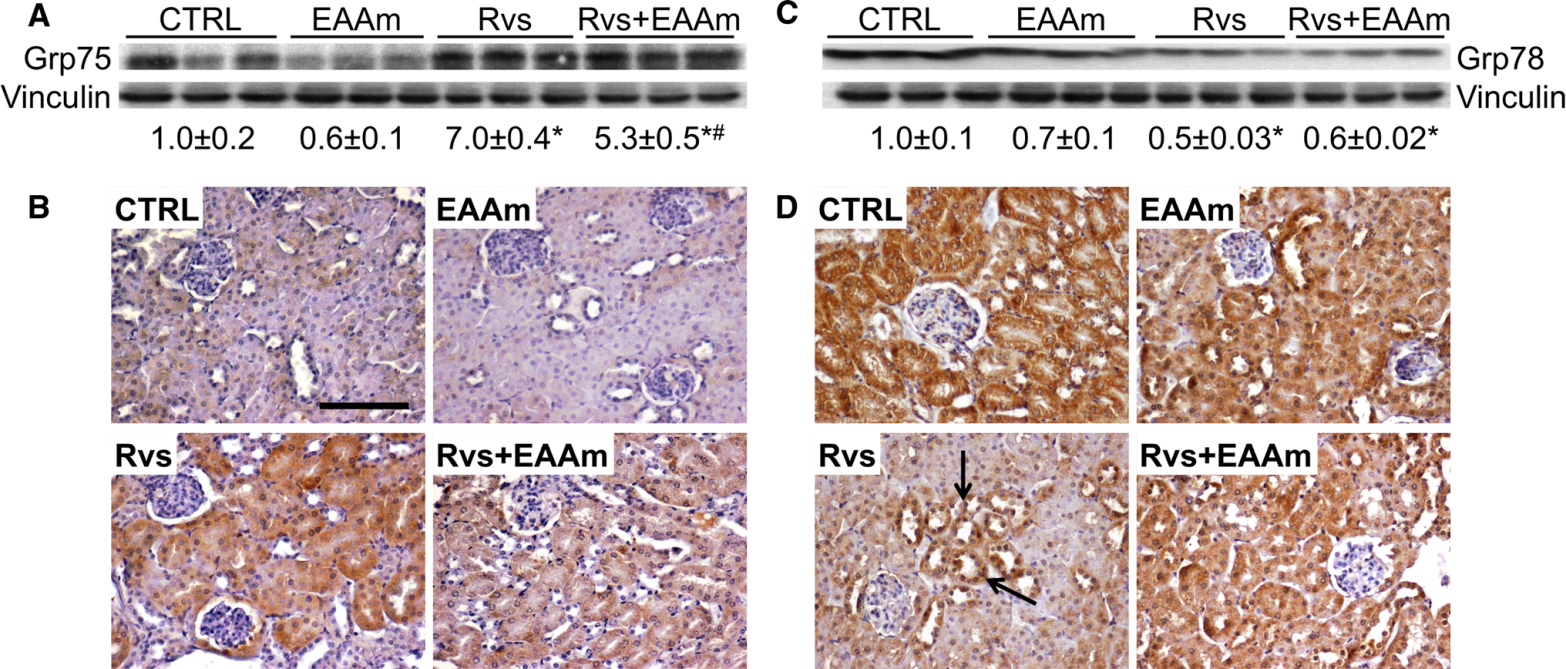
Renal ER stress is decreased by rosuvastatin and amino acids. **a**, **c** Grp75 and Grp78 protein levels were analyzed by immunoblot and are considered markers of ER stress. Relative values were detected by densitometric analysis, relative to vinculin levels, when control measurements were given a value of 1.0. The Western blot images are representative of separate experiments performed in 8–10 animals per group. Data are expressed as mean ± SEM. *p < 0.05 vs untreated (CTRL) mice. **b**, **d** Grp75 and Grp78 immunohistochemical staining. *EAAm* amino acid mixture enriched in essential amino acids, *Rvs* rosuvastatin. *Bar* 50 μm

S473-phosphorylated AKT was recently localized to the endoplasmic reticulum (ER) subcompartment, termed the mitochondria-associated ER membrane (MAM) (Betz et al. 2013). In mammalian MAM, the ER and mitochondria are physically tethered to each other by the inositol trisphosphate (IP3) receptor (IP3R)/glucose-regulated protein 75 (Grp75)/voltage-dependent anion-selective channel 1 (VDAC1) trimeric complex (Betz et al. 2013). Grp75 expression was therefore investigated. Rvs, in contrast to EAAm alone, markedly increased Grp75 protein levels in the kidneys of treated mice (Fig. 5a). This increased expression was not changed by EAAm supplementation. These results were confirmed by immunohistochemical analysis. Grp75 immunostaining was faint and uniformly distributed in tubular and glomerular cells of mice in the control and EAAm alone groups (Fig. 5b), but was strongly increased by Rvs, mainly in the proximal tubular cells with glomeruli only occasionally stained (Fig. 5b). Although Grp75 staining was less intense when Rvs was given with EAAm supplementation, it remained much higher than in untreated and EAAm-supplemented mice (Fig. 5b; Table 7).

Statins reduce ER stress in isolated cell systems, and glucose-regulated protein 78 (Grp78) is involved in the homeostatic response to cellular redox damage (Breder et al. 2010; Molins et al. 2010). Grp78 expression was thus examined in the kidneys of treated and untreated mice. Chronic exposure to Rvs reduced Grp78 protein levels (Fig. 5c), while EAAm treatment did not change either basal or Rvs-decreased Grp78 expression. Immunostaining clearly identified Grp78 in the tubular cells of untreated animals (Fig. 5d); however, Rvs treatment induced sparse and moderate cytoplasmic staining of glomerular cells, while the proximal tubules were intensely stained (Fig. 5d). EAAm only partly modified these staining patterns (Fig. 5d; Table 7). These findings suggest that Rvs positively modulates the expression of proteins controlling mitochondrial and ER physiology, and that EAAm supplementation does not play a relevant role in favoring these processes.

### Rosuvastatin and EAAm in combination promote an anti-apoptotic mechanism

**Fig. 6:**
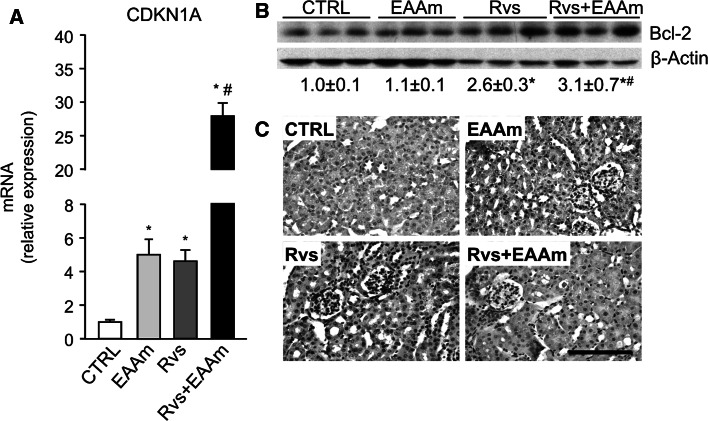
Rosuvastatin and amino acids stimulate an anti-apoptotic mechanism in mouse kidneys. **a** *CDKN1A* mRNA levels in kidney homogenates. **b** Bcl-2 protein levels. The Western blot images are representative of separate experiments performed in 8–10 animals per group. Data are expressed as mean ± SEM. *p < 0.05 vs untreated (CTRL) mice; ^#^ *p* < 0.05 vs Rvs-treated mice. **c** Bcl-2 immunohistochemical analysis. *EAAm* amino acid mixture enriched in essential amino acids, *Rvs* rosuvastatin. *Bar* 50 μm

Mitochondria and ER are involved in the apoptotic process, as are AKT, Grp75 and Grp78 (Tabas and Ron 2011; Logue et al. 2013). Rvs exerts a protective effect on injured podocytes through a p21-dependent anti-apoptotic pathway (Cormack-Aboud et al. 2009), and cytoplasmic p21 accumulation, due to AKT-dependent phosphorylation of nuclear p21, promotes a Bcl-2-dependent anti-apoptotic process (Vincent et al. 2012). For these reasons, the mRNA levels of *CDKN1A*, the gene encoding p21, were analyzed. Both Rvs and EAAm increased *CDKN1A* expression in the kidneys of treated mice (Fig. 6a). Rvs and EAAm in combination showed a strong synergistic effect on *CDKN1A* mRNA levels (Fig. 6a). Bcl-2 protein levels were increased by Rvs, but not by EAAm alone; however, the combination of Rvs and EAAm promoted higher Bcl-2 levels than Rvs alone (Fig. 6b). Immunohistochemical analysis showed faint Bcl-2 staining in all tubular cells in the untreated group, whereas Bcl-2 expression in tubular cells was increased in the kidneys of both Rvs- and EAAm-treated animals (Fig. 6c; Table 7). Bcl-2 staining was uniformly present in proximal tubular cells and was more marked in the Rvs plus EAAm group than in the Rvs group (Fig. 6c; Table 7).

## Discussion

The present results show that long-term treatment with Rvs, at a dose comparable to that normally used in clinical trials to lower blood cholesterol levels, promotes the expression of markers of mitochondrial biogenesis in the kidneys of young mice. In particular, ultrastructural analysis identifies abundant and elongated mitochondria around the nuclei of cortical proximal tubular cells in mice treated with Rvs. Multiform cristae and electron-dense granules in mitochondrial matrix are evident, suggesting either intense functional activity or calcium accumulation within the organelles (Khraiwesh et al. 2013). The protein levels of SIRT1 and PGC-1α, two master regulators of mitochondrial biogenesis, and COX IV, an OXPHOS respiratory protein, are increased by Rvs in the kidneys. Dietary supplementation with EAAm, which modifies the plasma amino acid profile, potentiates these effects. Recent results have suggested that SIRT1 in proximal tubules protects against albuminuria in diabetes by maintaining nicotinamide mononucleotide concentrations around glomeruli, thus influencing podocyte function (Hasegawa et al. 2013).

Previously, statins were reported to increase NO bioavailability in patients with hypercholesterolemia (John et al. 2001) and to exert beneficial effects on the activation and expression of eNOS in hypertensive rats (Kishi et al. 2008; Ohkawara et al. 2010). Moreover, Kureishi et al. (2000) demonstrated that simvastatin induces AKT-regulated eNOS activation (via Ser1177 phosphorylation), which increases NO production and consequently promotes endothelial cell survival. Similarly, EAAm was reported to promote mitochondrial biogenesis in cardiac and skeletal muscle by promoting eNOS activity (D’Antona et al. 2010) and AKT phosphorylation (Flati et al. 2010). Accordingly, the present results demonstrate that Rvs induces AKT phosphorylation at Ser473 and increases eNOS phosphorylation at Ser1177 in mouse kidney. Moderate eNOS immunostaining is uniformly distributed in cortical tubular cells and is evident in untreated and Rvs-treated or EAAm-supplemented mice. However, simultaneous administration of Rvs and EAAm markedly increases eNOS staining in the kidneys, particularly in the proximal tubules.

Controversial recent evidence suggests that statins may both activate or suppress the mTOR pathway in cancer or non-cancer cells (Roudier et al. 2006; Finlay et al. 2007). To this effect, Woodard et al. (2008) showed that fluvastatin induced apoptosis potently and limited the proliferation of renal cell carcinoma (RCC) cells in vitro; these effects were mediated by the suppression of AKT phosphorylation/activation, resulting in inhibition of mTOR and p70 S6 kinase. In the current study, S6 phosphorylation was not altered by Rvs and EAAm, alone or in combination. This result is consistent with a previous study in which dietary leucine supplementation did not modify the mTOR signaling in the kidney of neonatal pigs (Suryawan et al. 2012). Together these findings support a direct role for statins and amino acids in modulating the eNOS-dependent NO signaling pathway in renal tubular and endothelial cells through an AKT-dependent mTOR-independent pathway. In particular, the EAAs have been shown to increase synthesis of arginine through reinforcement of the anaplerotic export of aspartate, necessary for recycling of citrulline to arginine by arginino-succinate synthetase. Complex mechanisms, including the stimulatory effect of Rvs on eNOS activity and increased arginine availability from EAAm supplementation, may contribute to the processes observed in the present work.

Considering the positive effect of NO on mitochondrial biogenesis and function (Nisoli et al. 2003, 2004; Nisoli and Carruba 2006), the present results imply that Rvs, particularly when combined with EAAm, may have relevant beneficial effects on kidneys, and particularly on the proximal tubular cells. Notably, these cells do not use glucose for their energy production (Balaban and Mandel 1988) but rely primarily on fatty acid oxidation. Fatty acid oxidation is mostly performed by mitochondria, which therefore have a central role in these cells. Mitochondrial production of ATP, which is essential for generating the energy-dependent ion gradients that drive renal tubular reabsorption, is impaired in acute kidney injury (Hall et al. 2011). This can cause massive and life-threatening losses of fluids, electrolytes, and low-molecular weight nutrients, a dysfunction known as renal Fanconi’s syndrome (Che et al. 2014). Recently, a rare mutation was identified in autosomal dominant Fanconi’s syndrome that creates a new mitochondrial targeting motif in the N-terminal portion of enoyl-CoA hydratase/3-hydroxyacyl CoA dehydrogenase (EHHADH) (Klootwijk et al. 2014). EHHADH is involved in peroxisomal oxidation of fatty acids and is expressed in proximal tubules. Studies of proximal tubular cells expressing mutant EHHADH revealed that mistargeting of a peroxisomal protein to mitochondria impaired mitochondrial oxidative phosphorylation, with marked defects in the transport of fluids and glucose across the epithelium, indicative of the central role of mitochondria in proximal tubular function (Klootwijk et al. 2014). Although hyperaminoaciduria is a hallmark of the condition (Baum 1998), it is tempting to speculate that Rvs and particularly Rvs in combination with EAAm could have beneficial effects on mitochondrial function in the renal tubular cells impaired in Fanconi’s syndrome.

The present findings suggest that Rvs modulates the physiological relationship between the mitochondria and the ER. Rvs markedly increased the expression of Grp75 in the proximal tubular and glomerular cells. Grp75 is a component of a trimeric complex (IP3-Grp75-VDAC1) that regulates physical tethering of the ER and mitochondria at MAMs (Betz et al. 2013), together with mitofusins 1 and 2 (Twig et al. 2008). The ER–mitochondria tethering complex affects mitochondrial dynamics and function. In particular, association with the ER forms microdomains on the outer mitochondrial membrane. These sites are enriched in mitochondrial division components, such as dynamin-related guanosine triphosphatases 1 (DRP1) and membrane protein mitochondrial fission factor (Mff) (Friedman et al. 2011; Youle and van der Bliek 2012). Under stress conditions, the microdomains may recruit and regulate the activation of pro-apoptotic Bcl-2 proteins such as Bax, which promote permeabilization of the mitochondrial outer membrane and the release of death mediators such as Cyt c (Hoppins and Nunnari 2012). Thus, the ER–mitochondria contact points regulate apoptosis under particular conditions, in addition to modulating lipid transfer and calcium flux between the organelles. These processes are essential for mitochondrial function and ER homeostasis. Diverse evidence supports the effect of statins in reducing ER stress, and the role of Grp78, an ER stress sensor, in the homeostatic response to cellular redox damage has been explored (Breder et al. 2010; Molins et al. 2010). In the present work, long-term treatment with Rvs reduces Grp78 protein levels in kidney homogenates. This occurs specifically in glomerular cells, in contrast to the proximal tubular cells where Grp78 was accumulated. These findings suggest that Rvs can communicate diverse survival messages to different kidney cell types.

Rvs was previously found to protect injured podocytes through a p21-dependent anti-apoptotic pathway (Cormack-Aboud et al. 2009). Accordingly, the present results show that the anti-apoptotic Bcl-2 protein levels are increased by Rvs, alone and in combination with EAAm. Immunostaining confirms that Bcl-2 is accumulated in the tubular cells. Vincent et al. (2012) have recently demonstrated that cytoplasmic p21 accumulation, resulting from AKT-dependent phosphorylation of nuclear p21, promotes a Bcl-2-dependent anti-apoptotic process in cancer cells. Accordingly, both Rvs and EAAm here increase *CDKN1A* expression in the kidneys of treated mice, and this effect is more marked with Rvs and EAAm in combination.

These results are consistent with recent studies showing that both Rvs treatment and EAAm supplementation had beneficial effects on kidneys in rodents (Corsetti, et al. 2010) and humans (Vidt et al. 2006; Bolasco et al. 2011). Although data on the potential renal toxicity of high-potency statins are controversial (Tiwari 2006; Golomb and Evans 2008; Dodiya et al. 2011), two meta-analyses showed that statin therapy is safe and effective in preventing mortality and major cardiovascular events in patients with non-dialysis-dependent chronic kidney disease (CKD) (Upadhyay et al. 2012, 2013; Palmer et al. 2012). However, these studies provide very limited evidence to support the use of statins in patients on dialysis, and statin therapy was not found to be effective in reducing the risk of kidney failure or decline in kidney function (Upadhyay 2013). Conversely, a recent pilot study conducted in 15 CKD patients on hemodialysis treatment for at least 6 months has shown that 3 month EAAm supplementation increased serum albumin and total proteins, with reduced levels of inflammatory markers and improved anemia (Bolasco et al. 2011). Although these effects were studied in a small group of patients and might be due to ameliorated nutrient intake besides that of the amino acid mixture, EAAm supplementation might improve the efficacy of statin therapy in dialysis-dependent subjects.

## Conclusions

The present results support the favorable effects of a high-potency statin, like Rvs, on the morpho-functional aspects of mouse kidney. In particular, long-term treatment with Rvs supports mitochondrial function, probably through the AKT-eNOS signaling pathway, and regulates survival of proximal tubular cells. An original formula of essential amino acids enhances the statin’s effects. This provides mechanistic confirmation of recent clinical evidence in kidney disease and suggests the potential use of essential amino acid supplementation in conjunction with statins in patients with renal disorders characterized by tubular cell dysfunction.

## Abbreviations

p-AKT: Phosphorylated protein kinase B
Bcl-2: B-cell lymphoma 2
CDKN1A: Cyclin-dependent kinase inhibitor 1 (p21)
COX IV: Cytochrome c oxidase
Cyt c: Cytochrome c
EAAm: Essential amino acid mixture
p-eNOS: Phosphorylated endothelial nitric oxide synthase
ER: Endoplasmic reticulum
GAPDH: Glyceraldehyde 3-phosphate dehydrogenase
Grp75: Glucose-regulated protein 75
Grp78: Glucose-regulated protein 78
mtDNA: Mitochondrial DNA
NRF-1: Nuclear respiratory factor-1
OXPHOS: Oxidative phosphorylation
PGC-1α: Peroxisome proliferator-activated receptor γ coactivator-1 α
Tfam: Mitochondrial transcription factor A
Rvs: Rosuvastatin

## References

[CR1] Andrew PJ, Myer B (1999). Enzymatic function of nitric oxide synthases. Cardiovasc Res.

[CR2] Aquilani R, Viglio S, Iadarola P, Guarnaschelli C, Arrigoni N, Fugazza G, Catapano M, Boschi F, Dossena M, Pastoris O (2000). Peripheral plasma amino acid abnormalities in rehabilitation patients with severe brain injury. Arch Phys Med Rehabil.

[CR3] Bachmanov AA, Reed DR, Beauchamp GK, Tordoff MG (2002). Food intake, water intake, and drinking spout side preference of 28 mouse strains. Behav Genet.

[CR4] Bae E, Kim I, Park J, Ma S, Lee J, Kim S (2010). Renoprotective effect of rosuvastatin in DOCA-salt hypertensive rats. Nephrol Dial Transplant.

[CR5] Balaban RS, Mandel LJ (1988). Metabolic substrate utilization by rabbit proximal tubule: an NADH fluorescence study. Am J Physiol.

[CR6] Baum M (1998). The Fanconi syndrome of cystinosis: insights into the pathophysiology. Pediatr Nephrol.

[CR7] Betz C, Stracka D, Prescianotto-Baschong C, Frieden M, Demaurex N, Hall MN (2013). mTOR complex 2-AKT signaling at mitochondria-associated endoplasmic reticulum membranes (MAM) regulates mitochondrial physiology. Proc Natl Acad Sci USA.

[CR8] Bolasco P, Caria S, Cupisti A, Secci R, Dioguardi FS (2011). A novel amino acids oral supplementation in hemodialysis patients: a pilot study. Ren Fail.

[CR9] Breder I, Coope A, Arruda AP, Razolli D, Milanski M, Dorighello GG, de Oliveira HC, Velloso LA (2010). Reduction of endoplasmic reticulum stress-a novel mechanism of action of statins in the protection against atherosclerosis. Atherosclerosis.

[CR10] Bussolati B, Deregibus MC, Fonsato V, Doublier S, Spatola T, Procida S, Di Carlo F, Camussi G (2005). Statins prevent oxidized LDL-induced injury of glomerular podocytes by activating the phosphatidylinositol 3-kinase/AKT-signaling pathway. J Am Soc Nephrol.

[CR11] Cano NJ, Fouque D, Leverve XM (2006). Application of branched-chain amino acids in human pathological states: renal failure. J Nutr.

[CR12] Che R, Yuan Y, Huang S, Zhang A (2014). Mitochondrial dysfunction in the pathophysiology of renal diseases. Am J Physiol Renal Physiol.

[CR13] Cormack-Aboud FC, Brinkkoetter PT, Pippin JW, Shankland SJ, Durvasula RV (2009). Rosuvastatin protects against podocyte apoptosis in vitro. Nephrol Dial Transplant.

[CR14] Corsetti G, Rezzani R, Rodella L, Bianchi R (1998). Ultrastructural study of the alterations in spinal ganglion cells of rats chronically fed on ethanol. Ultrastruct Pathol.

[CR15] Corsetti G, Stacchiotti A, D’Antona G, Nisoli E, Dioguardi F, Rezzani R (2010). Supplementation with essential amino acids in middle age maintains the healthy of rat kidney. Int J Immunopathol Pharmacol.

[CR16] Corsetti G, Stacchiotti A, Tedesco L, D’Antona G, Pasini E, Dioguardi FS, Nisoli E, Rezzani R (2011). Essential amino acid supplementation decreases liver damage induced by chronic ethanol consumption in rats. Int J Immunopathol Pharmacol.

[CR17] Corsetti G, Pasini E, Ferrari-Vivaldi M, Romano C, Bonomini F, Tasca G, Dioguardi FS, Rezzani R, Assanelli D (2012). Metabolic syndrome and chronic simvastatin therapy enhanced human cardiomyocyte stress before and after ischemia-reperfusion in cardio-pulmonary bypass patients. Int J Immunopathol Pharmacol.

[CR18] D’Antona G, Ragni M, Cardile A, Tedesco L, Dossena M, Bruttini F, Caliaro F, Corsetti G, Bottinelli R, Carruba MO, Valerio A, Nisoli E (2010). Branched-chain amino acid supplementation promotes survival and supports cardiac and skeletal muscle mitochondrial biogenesis in middle-aged mice. Cell Metab.

[CR19] Dayan D, Hiss Y, Hirshberg A, Bubis J, Wolman M (1989). Are the polarization colors of picrosirius red-stained collagen determined only by the diameter of the fibers?. Histochemistry.

[CR20] De Pinieux G, Chariot P, Ammi-Saïd M, Louarn F, Lejonc JL, Astier A, Jacotot B, Gherardi R (1996). Lipid-lowering drugs and mitochondrial function: effects of HMG-CoA reductase inhibitors on serum ubiquinone and blood lactate/pyruvate ratio. Br J Clin Pharmacol.

[CR21] Dodiya H, Jain M, Goswami S (2011). Renal toxicity of lisinopril and rosuvastatin, alone and in combination, in Wistar rats. Int J Toxicol.

[CR22] Enomoto S, Sata M, Fukuda D, Nakamura K, Nagai R (2009). Rosuvastatin prevents endothelial cell death and reduces atherosclerotic lesion formation in ApoE-deficient mice. Biomed Pharmacother.

[CR23] Finlay GA, Malhowski AJ, Liu Y, Fanburg BL, Kwiatkowski DJ, Toksoz D (2007). Selective inhibition of growth of tuberous sclerosis complex 2 null cells by atorvastatin is associated with impaired Rheb and Rho GTPase function and reduced mTOR/S6 kinase activity. Cancer Res.

[CR24] Fiore M, Jimenez P, Cremonezzi D, Juncos L, Garcia N (2011). Statins reverse renal inflammation and endothelial dysfunction induced by chronic high salt intake. Am J Physiol Renal Physiol.

[CR25] Flati V, Caliaro F, Speca S, Corsetti G, Cardile A, Nisoli E, Bottinelli R, D’ Antona G (2010). Essential amino acids improve insulin activation of AKT/MTOR signaling in soleus muscle of aged rats. Int J Immunopathol Pharmacol.

[CR26] Friedman JR, Lackner LL, West M, DiBenedetto JR, Nunnari J, Voeltz GK (2011). ER tubules mark sites of mitochondrial division. Science.

[CR27] Gambelli S, Dotti MT, Malandrini A, Mondelli M, Stromillo ML, Gaudiano C, Federico A (2004). Mitochondrial alterations in muscle biopsies of patients on statin therapy. J Submicrosc Cytol Pathol.

[CR28] Golomb BA, Evans MA (2008). Statin adverse effects: a review of the literature and evidence for a mitochondrial mechanism. Am J Cardiovasc Drugs.

[CR29] Hall AM, Hendry BM, Nitsch D, Connolly JO (2011). Tenofovir-associated kidney toxicity in HIV-infected patients: a review of the evidence. Am J Kidney Dis.

[CR30] Hasegawa K, Wakino S, Simic P, Sakamaki Y, Minakuchi H, Fujimura K, Hosoya K, Komatsu M, Kaneko Y, Kanda T, Kubota E, Tokuyama H, Hayashi K, Guarente L, Itoh H (2013). Renal tubular Sirt1 attenuates diabetic albuminuria by epigenetically suppressing Claudin-1 overexpression in podocytes. Nat Med.

[CR31] Hoppins S, Nunnari J (2012). Mitochondrial dynamics and apoptosis—the ER connection. Science.

[CR32] Ito D, Ito O, Mori N, Muroya Y, Cao PY, Takashima K, Kanazawa M, Kohzuki M (2010). Atorvastatin upregulates nitric oxide synthases with Rho-kinase inhibition and AKT activation in the kidney of spontaneously hypertensive rats. J Hypertens.

[CR33] John S, Delles C, Jacobi J, Schlaich MP, Schneider M, Schmitz G, Schmieder RE (2001). Rapid improvement of nitric oxide bioavailability after lipid-lowering therapy with cerivastatin within two weeks. J Am Coll Cardiol.

[CR34] Kano K, Hirata Y, Emory T, Ohta K, Eguci S, Imai T, Marumo F (1992). l-arginine infusion induces hypotension and diuresis/natriuresis with concomitant increased urinary excretion of nitrite/nitrate and cyclic GMP in humans. Clin Exp Pharmacol Physiol.

[CR35] Khraiwesh H, Lopez-Dominguez J, Lopez-Lluch G, Navas P, de Cabo R, Ramsey J, Villalba J, Gonzalez-Reyes J (2013). Alterations of ultrastructural and fission/fusion markers in hepatocyte mitochondria from mice following calorie restriction with different dietary fats. J Gerontology A Biol Sci Med Sci.

[CR36] Kishi T, Hirooka Y, Shimokawa H, Takeshita A, Sunagawa K (2008). Atorvastatin reduces oxidative stress in the rostral ventrolateral medulla of stroke-prone spontaneously hypertensive rats. Clin Exp Hypertens.

[CR37] Klootwijk ED, Reichold M, Helip-Wooley A, Tolaymat A, Broeker C, Robinette SL, Reinders J, Peindl D, Renner K, Eberhart K, Assmann N, Oefner PJ, Dettmer K, Sterner C, Schroeder J, Zorger N, Witzgall R, Reinhold SW, Stanescu HC, Bockenhauer D, Jaureguiberry G, Courtneidge H, Hall AM, Wijeyesekera AD, Holmes E, Nicholson JK, O’Brien K, Bernardini I, Krasnewich DM, Arcos-Burgos M, Izumi Y, Nonoguchi H, Jia Y, Reddy JK, Ilyas M, Unwin RJ, Gahl WA, Warth R, Kleta R (2014). Mistargeting of Peroxisomal EHHADH and Inherited Renal Fanconi’s Syndrome. New Engl J Med.

[CR38] Urquhart BL, zu Schwabedissen HEM, Schwarz UI, Lemke CJ, Leake BF, Kim RB, Tirona RG, Knauer MJ (2010). Human skeletal muscle drug transporters determine local exposure and toxicity of statins. Circ Res.

[CR39] Kureishi Y, Luo Z, Shiojima I, Bialik A, Fulton D, Lefer DJ, Sessa WC, Walsh K (2000). The HMG-CoA reductase inhibitor simvastatin activates the protein kinase AKT and promotes angiogenesis in normocholesterolemic animals. Nat Med.

[CR40] Larsen S, Stride N, Hey-Mogensen M, Hansen CN, Bang LE, Bundgaard H, Nielsen LB, Helge JW, Dela F (2013). Simvastatin effects on skeletal muscle: relation to decreased mitochondrial function and glucose intolerance. J Am Coll Cardiol.

[CR41] Lenaz G, Bovina C, D’Aurelio M, Fato R, Formiggini G, Genova ML, Giuliano G, Pich MM, Paolucci U, Castelli GP, Ventura B (2002). Role of mitochondria in oxidative stress and aging. Ann N Y Acad Sci.

[CR42] Logue SE, Cleary P, Saveljeva S, Samali A (2013). New directions in ER stress-induced cell death. Apoptosis.

[CR43] MacAllister R, Vallance P (1994). Nitric oxide in essential and renal hypertension. J Am Soc Nephrol.

[CR44] Mancini GB, Tashakkor AY, Baker S, Bergeron J, Fitchett D, Frohlich J, Genest J, Gupta M, Hegele RA, Ng DS, Pearson GJ, Pope J (2013). Diagnosis, prevention, and management of statin adverse effects and intolerance: canadian working group consensus update. Can J Cardiol.

[CR45] Mansi I, Frei CR, Pugh MJ, Makris U, Mortensen EM (2013). Statins and musculoskeletal conditions, arthropathies, and injuries. JAMA Intern Med.

[CR46] Molins B, Peña E, Padro T, Casani L, Mendieta C, Badimon L (2010). Glucose-regulated protein 78 and platelet deposition: effect of rosuvastatin. Arterioscler Thromb Vasc Biol.

[CR47] Nisoli E, Carruba MO (2006). Nitric oxide and mitochondrial biogenesis. J Cell Sci.

[CR48] Nisoli E, Clementi E, Paolucci C, Cozzi V, Tonello C, Sciorati C, Bracale R, Valerio A, Francolini M, Moncada S, Carruba MO (2003). Mitochondrial biogenesis in mammals: the role of endogenous nitric oxide. Science.

[CR49] Nisoli E, Falcone S, Tonello C, Cozzi V, Palomba L, Fiorani M, Pisconti A, Brunelli S, Cardile A, Francolini M, Cantoni O, Carruba MO, Moncada S, Clementi E (2004). Mitochondrial biogenesis by NO yields functionally active mitochondria in mammals. Proc Natl Acad Sci USA.

[CR50] Ohkawara H, Ishibashi T, Saitoh S, Inoue N, Sugimoto K, Kamioka M, Uekita H, Kaneshiro T, Ando K, Takuwa Y, Maruyama Y, Takeishi Y (2010). Preventive effects of pravastatin on thrombin-triggered vascular responses via AKT/eNOS and RhoA/Rac1 pathways in vivo. Cardiovasc Res.

[CR51] Palmer SC, Craig JC, Navaneethan SD, Tonelli M, Pellegrini F, Strippoli GF (2012). Benefits and harms of statin therapy for persons with chronic kidney disease: a systematic review and meta-analysis. Ann Intern Med.

[CR52] Pellegrino MA, Brocca L, Dioguardi F, Bottinelli R, D’Antona G (2005). Effects of voluntary wheel running and amino acid supplementation on skeletal muscle of mice. Eur J Physiol.

[CR53] Rajapaskse NW, Mattson DL (2013). Role of cellular L-arginine and nitric oxide production on renal blood flow and arterial pressure regulation. Curr Opin Nephrol Hypertens.

[CR54] Robles NR, Velasco J, Mena C, Polo J, Angulo E, Espinosa J (2013). Increased frequency of microalbuminuria in patients receiving statins. Clin Lipidol.

[CR55] Rondanelli M, Opizzi A, Antoniello N, Boschi F, Iadarola P, Pasini E, Aquilani R, Dioguardi FS (2011). Effect of essential amino acid supplementation on quality of life, amino acid profile and strength in institutionalized elderly patients. Clin Nutr.

[CR56] Rosenfeldt FL, Pepe S, Linnane A, Nagley P, Rowland M, Ou R, Marasco S, Lyon W, Esmore D (2002). Coenzyme Q10 protects the aging heart against stress: studies in rats, human tissues, and patients. Ann NY Acad Sci.

[CR57] Roudier E, Mistafa O, Stenius U (2006). Statins induce mammalian target of rapamycin (mTOR)-mediated inhibition of Akt signaling and sensitize p53-deficient cells to cytostatic drugs. Mol Cancer Ther.

[CR58] Schick BA, Laaksonen R, Frohlich JJ, Päivä H, Lehtimäki T, Humphries KH, Côté HC (2007). Decreased skeletal muscle mitochondrial DNA in patients treated with high-dose simvastatin. Clin Pharmacol Ther.

[CR59] Shillingford JM, Murcia NS, Larson CH, Low SH, Hedgepeth R, Brown N, Flask CA, Novick AC, Goldfarb DA, Kramer-Zucker A, Waltz G, Piontek KB, Germino GG, Weimbs T (2006). The mTOR pathway is regulated by polycystin-1, and its inhibition reverses renal cystinosis in polycystic kidney disease. Proc Natl Acad Sci USA.

[CR60] Silbernagl S (1988). The renal handling of amino acids and oligopeptides. Physiol Rev.

[CR61] Stacchiotti A, Volti GL, Lavazza A, Schena I, Aleo MF, Rodella L, Rezzani R (2011). Different role of Schisandrin B on mercury-induced renal damage in vivo and in vitro. Toxicology.

[CR62] Stein EA, Vidt DG, Shepherd J, Cain VA, Anzalone D, Cressman MD (2012). Renal safety of intensive cholesterol-lowering treatment with rosuvastatin: a retrospective analysis of renal adverse events among 40,600 participants in the rosuvastatin clinical development program. Atherosclerosis.

[CR63] Stone NJ, Robinson J, Lichtenstein AH, Bairey Merz CN, Lloyd-Jones DM, Blum CB, McBride P, Eckel RH, Schwartz JS, Goldberg AC, Shero ST, Gordon D, Smith SC Jr, Levy D, Watson K, Wilson PW. (2013) 2013 ACC/AHA Guideline on the Treatment of Blood Cholesterol to Reduce Atherosclerotic Cardiovascular Risk in Adults: A Report of the American College of Cardiology/American Heart Association Task Force on Practice Guidelines Circulation. doi:10.1016/j.jacc.2013.11.002 10.1016/j.jacc.2013.11.002 24239923

[CR64] Suryawan A, Torrazza RM, Gazzaneo MC, Orellana RA, Fiorotto ML, El-Kadi SW, Srivastana N, Nguyen HV, Davis TA (2012). Enteral leucine supplementation increases protein synthesis in skeletal and cardiac muscles and visceral tissues of neonatal pigs through mTORC1-dependent pathways. Pediatr Res.

[CR65] Susic D, Varagic J, Ahn J, Slama M, Frohlich E (2003). Beneficial pleiotropic vascular effects of rosuvastatin in two hypertensive models. J Am Coll Cardiol.

[CR66] Szeto HH, Liu S, Soong Y, Wu D, Darrah SE, Cheng FY, Zhao Z, Ganger M, Tow CY, Seshan SV (2011). Mitochondria-targeted peptide accelerates ATP recovery and reduce ischemic kidney injury. J Am Soc Nephrol.

[CR67] Tabas I, Ron D (2011). Integrating the mechanisms of apoptosis induced by endoplasmic reticulum stress. Nat Cell Biol.

[CR68] Tedesco L, Valerio A, Dossena M, Cardile A, Ragni M, Pagano C, Pagotto U, Carruba MO, Vettor R, Nisoli E (2010). Cannabinoid receptor stimulation impairs mitochondrial biogenesis in mouse white adipose tissue, muscle, and liver: the role of eNOS, p38 MAPK, and AMPK pathways. Diabetes.

[CR69] Tiwari A (2006). An overview of statin-associated proteinuria. Drug Discov Today.

[CR70] Trupp M, Zhu H, Wikoff WR, Baillie RA, Zeng Z-B, Karp PD, Fiehn O, Krauss RM, Kaddurah-Daouk R (2012). Metabolomics reveals amino acids contribute to variation in response to simvastatin treatment. PLoS One.

[CR71] Twig G, Elorza A, Molina AJ, Mohamed H, Wikstrom JD, Walzer G, Stiles L, Haigh SE, Katz S, Las G, Alroy J, Wu M, Py BF, Yuan J, Deeney JT, Corkey BE, Shirihai OS (2008). Fission and selective fusion govern mitochondrial segregation and elimination by autophagy. EMBO J.

[CR72] Upadhyay A (2013) Statins in chronic kidney disease: what do meta-analysis tell us? Clin Exp Nephrol [Epub ahead of print] 10.1007/s10157-013-0889-2 24158229

[CR73] Upadhyay A, Earley A, Lamont JL, Haynes S, Wanner C, Balk EM (2012). Lipid-lowering therapy in persons with chronic kidney disease: a systematic review and meta-analysis. Ann Intern Med.

[CR74] Valerio A, D’Antona G, Nisoli E (2011). Branched-chain aminoacids, mitochondrial biogenesis, and health span: an evolutionary perspective. Aging.

[CR75] van der Tol A, Van Biesen W, Van Laecke S, Bogaerts K, De Lombaert K, Warrinnier H, Vanholder R (2012). Statin use and the presence of microalbuminuria. Results from the ERICABEL trial: a non-interventional epidemiological cohort study. PLoS One.

[CR76] Verhulst A, Sayer R, De Broe M, D’Haese P, Brown C (2008). Human proximal tubular epithelium actively secretes but does not retain rosuvastatin. Mol Pharmacol.

[CR77] Vidt DG, Harris S, McTaggart F, Ditmarsch M, Sager PT, Sorof JM (2006). Effect of short-term rosuvastatin treatment on estimated glomerular filtration rate. Am J Cardiol.

[CR78] Vincent AJ, Ren S, Harris LG, Devine DJ, Samant RS, Fodstad O, Shevde LA (2012). Cytoplasmic translocation of p21 mediates NUPR1-induced chemoresistance: NUPR1 and p21 in chemoresistance. FEBS Lett.

[CR79] Woodard J, Sassano A, Hay N, Platanias LC (2008). Statin-dependent suppression of the Akt/mammalian target of rapamycin signaling cascade and programmed cell death 4 up-regulation in renal cell carcinoma. Clin Cancer Res.

[CR80] Youle RJ, van der Bliek AM (2012). Mitochondrial fission, fusion, and stress. Science.

[CR81] Zoncu R, Efeyan A, Sabatini DM (2011). mTOR: from growth signal integration to cancer, diabetes and ageing. Nat Rev Mol Cell Biol.

